# Cell Phones and *Acinetobacter* Transmission

**DOI:** 10.3201/eid1107.050221

**Published:** 2005-07

**Authors:** Abraham Borer, Jacob Gilad, Rozalia Smolyakov, Seada Eskira, Nechama Peled, Nurith Porat, Eytan Hyam, Ronit Trefler, Klaris Riesenberg, Francisc Schlaeffer

**Affiliations:** * Soroka University Medical Center and Ben-Gurion University of the Negev, Beer-Sheva, Israel

**Keywords:** cellular phone, Acinetobacter baumannii, resistance, transmission, risk factor, nosocomial

**To the Editor:** Nosocomial *Acinetobacter baumannii* is commonly acquired through cross-transmission because of its propensity to survive in the hospital environment and persistently contaminate fomites. Since cell phones are used increasingly by health personnel worldwide, we sought to determine their role in nosocomial transmission of multidrug-resistant (MDR) *A. baumannii*.

The study was conducted in a tertiary-care hospital in Israel, where MDR *Acinetobacter* spp. is endemic. Cell phones are used by personnel both for private communication and instead of traditional pagers. During 2002, 124 personnel (71 physicians, 54 nurses) were screened randomly for *Acinetobacter* spp. in a point-prevalence study; samples from hands of 119 personnel and 124 cell phones were cultured simultaneously for 2 months. Swabs from the back and sides of the cell phones were cultured. Cultures of hand samples were done by using the broth-bag technique (1).

To assess cross-transmission between hands, cell phones, and patients, we studied 2 additional *Acinetobacter* spp. culture cohorts, nosocomial blood isolates from 2000 to 2002, and axilla and groin *Acinetobacter* spp. skin colonization in an intensive care unit (ICU) during 2002. Cohorts represent wards in which 73% of study personnel worked.

Isolates were identified by the ID20NE system (bioMérieux, Marcy l'Etoile, France) without differentiation between *A. baumannii* and species 3 and 13TU. Antimicrobial susceptibility was determined for aminoglycosides, penicillins, cephalosporins, carbapenems, fluoroquinolones, tetracyclines, polymyxin E, and ampicillin/sulbactam by using disk diffusion according to Clinical and Laboratory Standards Institute guidelines (2). MDR was defined as resistance ≥3 different classes.

Genotypic analysis of isolates from all cohorts was performed using pulsed-field gel electrophoresis. Chromosomal DNA was digested with *ApaI* and analyzed by using a CHEF-DRIII apparatus (Bio-Rad Laboratories, Hercules, CA, USA). Strain relatedness was interpreted according to consensus (3). Isolates showing an identical banding pattern were considered indistinguishable, and those showing differences of 3 bands were considered closely related.

Study personnel were assigned to medical (22%), surgical (44%), pediatric (23%), and ICU (11%) wards. The respective contamination rate with *Acinetobacter* spp. was 27%, 7.4%, 7.4%, and 0% for cell phones and 24%, 22%, 14%, and 41% for personnel hands. Of 30 hand and 15 cell phone cultures positive for *Acinetobacter* spp., 17% and 20%, respectively, were MDR.

Both hand and cell phone cultures of 3 personnel were positive (unrelated strains). Cell phone and hand isolates exhibited substantial clonal diversity. *Acinetobacter* spp. transmission (including MDR strains) was documented between hands, as well as between cell phones and hands, of different persons (Figure, panel A). One clone, recovered from cell phones and hands of ICU personnel, was also involved in skin colonization of ICU patients (Figure, panel B) but was unrelated to blood isolates.

**Figure:**
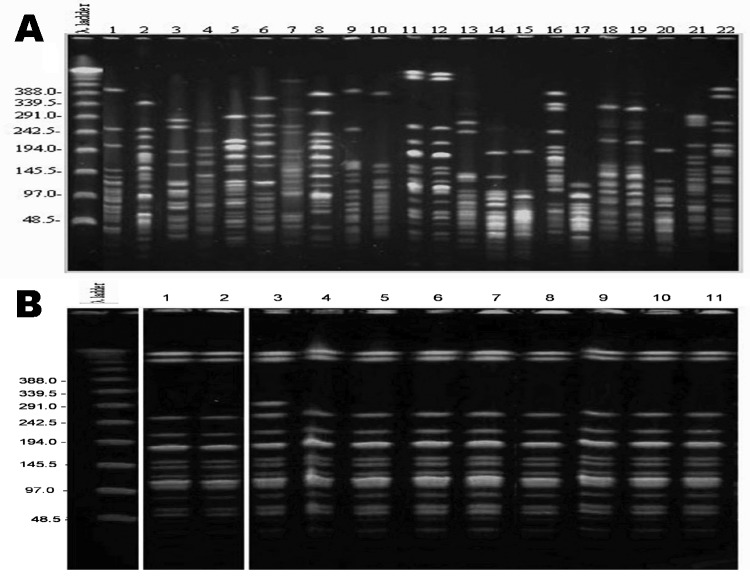
Pulsed-field gel electrophoresis of representative *Acinetobacter* strains. Twenty different clones (panel A) were recovered from cell phones (lanes no. 1, 3, 8–11) and hands of personnel (remaining lanes). Indistinguishable isolates were recovered from cellphones and hand cultures (lanes 11 and 12), and 2 hand cultures (lanes 18 and 19). Both pairs were obtained from different persons. Panel B shows a multidrug-resistant *Acinetobacter* spp. strain recovered from cell phones (lane 1), personnel hand cultures (lane 2), and patients with skin colonization (lanes 3–11). All isolates are indistinguishable except for no. 3, which is a closely related strain (demonstrating a 1-band difference). Unmarked lanes denote a molecular weight marker. Values on the left are in basepairs.

We found that a significant percentage of cell phones and hands were contaminated with MDR *Acinetobacter* spp. and that cross-contamination between hands, cell phones, and patients occurred. Co-contamination of hands and cell phones was found in only 10% of cases and may be explained by small sample size and that personnel were sampled only once. Higher co-contamination would likely have been found with repeated sampling.

The ability of *Acinetobacter* spp. to contaminate cell phones is not unexpected; it has been isolated from numerous sources in hospital environments in outbreak and nonoutbreak settings. Contamination and nosocomial transmission of pathogens by other electronic devices also has been demonstrated; a contaminated personal computer has been implicated in transmission of methicillin-resistant *Staphylococcus aureus* to a nurse. Computer keyboards have been contaminated with staphylococci and *Pseudomonas* spp (4). Keyboards also have been implicated in nosocomial *A. baumannii* infection in burn units and ICUs (5) and have been contaminated with enterococci and *Enterobacter* spp with a genetically identical methicillin-resistant *S. aureus* strain (6).

Stationary phones may also harbor pathogens; stationary phones in a daycare facility were contaminated with rotavirus (7), and home phones were contaminated with enteroviral DNA (8). In the hospital, ≤47% of stationary phones were contaminated with pathogenic microbes (9). Hand-to-mouth transfer of microbes was documented after contaminated fomites were handled during casual activities, with the highest transfer efficiency noted with stationary phone receivers (10).

Thus, cell phones may have a notable role in the nosocomial transmission of MDR microbes to patients. Cell phones are particularly problematic compared to stationary devices and may facilitate intra- and inter-ward (and perhaps inter-hospital) transmission. Additionally, the potential for nosocomial transmission of MDR pathogens by other electronic devices, such as handheld computers or personal digital assistants, with bedside applications, should be recognized.

Since restriction or even prohibition of such devices may prove impractical, strategies for preventing nosocomial transmission in this context are needed, especially given the risk of continuing contamination through repeated hand–cell phone contact. Such strategies should target behavioral controls of personnel (enforcing infection control precautions), environmental disinfection, and ultimately, optimal disinfection methods that will prevent contamination without damaging these sensitive electronic devices.

This work has been presented in part at the 43rd Interscience Conference on Antimicrobial Agents and Chemotherapy, Chicago, IL, USA, September 2003.
